# The Association Between Consumption of 100% Fruit Juice and Risk of Age-Related Macular Degeneration: Data From the National Health and Nutrition Examination Survey Database

**DOI:** 10.3389/fnut.2022.812476

**Published:** 2022-04-21

**Authors:** Mi Song, Baihua Chen

**Affiliations:** ^1^Department of Ophthalmology, The Second Xiangya Hospital of Central South University, Changsha, China; ^2^Hunan Clinical Research Center of Ophthalmic Disease, Changsha, China

**Keywords:** association, 100% fruit juice, pure juice, age-related macular degeneration, NHANES

## Abstract

Age-related macular degeneration (AMD) is the main irreversible blindness disease worldwide. The current study aimed to investigate whether the consumption of 100% fruit juice increases the risk of age-related macular degeneration and find approaches to prevent and reduce the development of age-related macular degeneration from the aspect of dietary habits. A cross-sectional clinical study design was adopted. We screened participants from the 2005 to 2006 NHANES database. The logistic regression model was used to evaluate the relationship between 100% fruit juice consumption and advanced AMD and to adjust variables such as demographics, general health status, body mass index (BMI), health-related behaviors, systemic complications, and ophthalmic complications. The results show that 100% fruit juice consumption did not affect early AMD and any AMD. High consumers of 100% fruit juice are more likely to develop advanced age-related macular degeneration than those who never drink 100% fruit juice.

## Introduction

Age-related macular degeneration (AMD) is the leading cause of visual impairment worldwide, and it is clinically divided into early and late AMD (1). The clinical features of early AMD are moderate drusen or retinal pigment abnormalities, and advanced AMD includes wet neovascular AMD and dry regional atrophy, which are related to visual impairment (1, 2). Globally, the prevalence of AMD in people aged 45–85 years is 8.69% (95% CI, 4.26–17.40%), and the prevalence of early and late AMD is 8.01% (95% CI, 3.98–15.49%), 0.37% (0.18–0.77%) (3). The 2011 epidemiological survey showed that the estimated prevalence of any AMD in the United States was 6.5% (95% CI, 5.5–7.6%), and the estimated prevalence of advanced AMD was 0.8% (95% CI, 0.5–1.3%) (4). Another United States survey stated that the incidence of advanced AMD is 1.47% (95%CI, 1.38–1.55%) among people ≥40 years of age (5). The pathogenesis of AMD involves disorders of oxidative stress, complement, lipids, angiogenesis, inflammation, and extracellular matrix pathways (1). The risk factors mainly include aging, genetic factors, and non-genetic risk factors (smoking, obesity, cardiovascular disease, lutein, omega 3 fatty acids and antioxidant zinc, vitamin A, C, E, and other insufficient intakes) (2). Although intravitreal injection of anti-VEGF factors (bevacizumab, ranibizumab, and aflibercept) effectively treats neovascular AMD (6, 7), the pathogenesis of AMD is not completely elucidated. Currently, there is no effective treatment for dry AMD. The occurrence and development of AMD are still a huge challenge (1).

There is still controversy about whether drinking 100% fruit juice is beneficial or harmful to human health. Some studies oppose the consumption of 100% fruit juice because it is found that the consumption of 100% fruit juice is associated with dental caries in children, weight gain of children and adults (8–13). Some experts recommend limiting 100% fruit juice consumption since it lacks dietary fiber and induces excessive natural sugar and energy intake (14). Another study stated that there is no clear evidence that 100% fruit juice consumption is related to diabetes, hypertension, and hyperlipidemia (15).

The National Health and Nutrition Examination Survey (NHANES) is carried out by the Centers for Disease Control and Prevention (CDC) to examine the health and nutritional status of the entire U.S. population. It collects data on health and disease conditions, drug use, nutritional intake, and physical examinations. The data published by NHANES from 2005 to 2006 can provide detailed information on the frequency of dietary use by participants, as well as data on AMD staging for participants aged 40 and above based on fundus photographs. More detailed information can be found on the NHANES official website.

There is a lack of study on the relationship between 100% fruit juice and AMD. Whether 100% juice consumption increases the risk of AMD is unclear. Exploring the relationship between 100% fruit juice intake and AMD may provide the evidence-based basis for dietary recommendations to help patients and doctors better manage AMD. In the current study, by analyzing the data of the NHANES database from 2005 to 2006, we hypothesized that the high consumption of 100% fruit juice is related to the higher risk of advanced AMD.

## Materials and Methods

### National Health and Nutrition Examination Survey

We used publicly available data from 2005 to 2006 NHANES. The NHANES project began in the early 1960s to provide data on Americans’ health and nutritional status. The survey collects about 5,000 non-institutionalized samples of American non-institutionalized peoples in stratified and multi-stages each year and weights the American population. It mainly includes demographic data, dietary data, physical examination data, laboratory data, and questionnaire survey data. The project passed the ethical approval of a human subject’s board and obtained written informed consent from all participants. All procedures follow the principles of the Declaration of Helsinki.

### Inclusion and Exclusion of Participants

All participants of the 2005–2006 NHANES project who were ≥40 years old and did not meet any exclusion criteria were included. Exclusion criteria were: (1) Those who are less than 40 years old (NHANES does not take retinal images for participants younger than 40 years old). (2) Those who lack light perception in both eyes, have eye infections, or wear goggles on both eyes. (3) Those with missing or unusable retinal images due to unrecognizable retinal images. (4) Those with missing data on 100% fruit juice drinking.

### Age-Related Macular Degeneration

The main outcome variable is whether to have advanced AMD. AMD staging is based on retinal images. Each eye obtains two 45-degree digital images of the retina under the small pupil with the macula and the optic nerve as the center. Then, the University of Wisconsin-Madison (University of Wisconsin-Madison, Madison, WI, United States) graders used a standardized method to grade the retinal images. The scoring team consists of one junior scoring coordinator, two junior raters, and six detailed raters. First, EyeQ Lite was used for semi-quantitative evaluation of digital retinal images, and a preliminary scoring coordinator reviewed important pathological images. Then, the retinal image is further graded by at least one primary grader and one detail grader. If the two raters disagree, then the third rater will classify the retinal image. If two of the three scorers disagree, then the judges will make the final decision. Early AMD retinal image features: soft drusen combined with grid-like degeneration areas greater than 500 μm in diameter and abnormal pigmentation or soft drusen and central pigment abnormality without signs of advanced AMD. Image features of advanced AMD: geographic atrophy, retinal pigment epithelial detachment, subretinal hemorrhage, fibrous scar, or neovascularization. When both eyes have different degrees of AMD, this study uses the AMD staging of the worse eye.

### 100% Fruit Juice Consumption

The main exposure variable is 100% fruit juice consumption. 100% fruit juice consumption data were acquired from the FFQ Food Frequency Questionnaire in the NHANES database, jointly developed by the National Institutes of Health (NIH) and the National Cancer Institute (NCI). FFQ was added to the 2003–2004 NHANES to determine food and food group consumption patterns in the last year. The same FFQ was used in NHANES from 2005 to 2006. NHANES provides 100% fruit juice consumption information in the diet data module. During the interview, participants were asked, “how often did you drink other 100% fruit juice or 100% fruit juice mixtures (such as pineapple, prune, or others)?” The answers were: never, 1 time per month or less, 2–3 times per month, 1–2 times per week, 3–4 times per week, 5–6 times per week, 1 time per day, 2–3 times per day, 4–5 times per day, 6 or more times per day. In this study, participants were divided into a never-consuming group, a moderate-consuming group: 1 time per month or more and less than 1 time per day, and a high-consuming group: 1 time per day or more. A study on 100% fruit juice used the same grouping method: the 100% fruit juice consumption data was transformed into <1 serving daily and ≥1 serving daily, as well as into categories of no juice (13).

### Covariates

Potential confounding factors include demographic data: gender, age, race, education, income to poverty ratio; health status; BMI; health-related behaviors: smoking, drinking; systemic disease comorbidities: diabetes, hypertension, hyperlipidemia Symptoms; ocular complications: glaucoma, cataract surgery history, and diabetic retinopathy. Information about gender, age, race, education, income, poverty ratio come from the NHANES demographic data module. BMI and diabetic retinopathy come from the NHANES inspection data module. The remaining variables are from the NHANES questionnaire data module. Smoking status: used tobacco/nicotine last 5 days? Alcohol usage: had at least 12 alcohol drinks/1 year? Self-reported diseases are determined by answering the following questions in the affirmative. Hypertension: “Did the doctor or other health experts tell you that you have high blood pressure?” Hyperlipidemia: “Did the doctor or other health experts tell you that your blood cholesterol level is high?” Diabetes: “Doctor or other health experts told you that you have diabetes or diabetes)?” Glaucoma: “Have you or Has SP ever been told by an ophthalmologist that you have or he/she have glaucoma, sometimes called high pressure in your or his/her eyes?” History of cataract surgery: “Have you/Has SP ever had a cataract operation?”

### Statistics Analysis

This study conducted data analysis according to the analysis guidelines compiled by the National Center for Health Statistics of the United States, and the analysis software used included R version R-3.6.3: http://www.R-project.org and EMPowerStats software: http://www.empowerstats.com. The significance level was 0.05. Due to the data missing in the covariable, we used multiple interpolation methods to fill it, including five sets of data after interpolation and one set of original data. A binomial logistic regression model was used to determine the relationship between 100% fruit juice consumption and advanced AMD. According to STROBE Guide (16), we built three models: Model 1, without adjusting any covariates; Model 2, adjusting for age, sex, and race; and Model 3, the covariables in Table 1 were all adjusted.

**TABLE 1:** Socio-demographic and clinical characteristics of participants by age-related macular degeneration (AMD) status.

	AMD status			
	
Characteristics	No ARM	Early ARM	Late ARM	*P*-value
N	1,425	115	16	
Age (year)	59.1 ± 12.6	70.8 ± 11.4	78.1 ± 7.4	<0.001
PIR	2.9 ± 1.6	2.5 ± 1.5	2.8 ± 1.5	0.086
Body mass index (kg/m^2^)	29.3 ± 7.0	28.8 ± 5.1	24.6 ± 4.0	0.024
100% fruit juice consumption				0.048
Never	506 (35.5%)	46 (40.0%)	3 (18.8%)	
Moderate	854 (59.9%)	62 (53.9%)	10 (62.5%)	
High	65 (4.6%)	7 (6.1%)	3 (18.8%)	
Gender				0.054
Male	718 (50.4%)	71 (61.7%)	7 (43.8%)	
Female	707 (49.6%)	44 (38.3%)	9 (56.2%)	
RACE				0.054
Mexican American	195 (13.7%)	16 (13.9%)	0 (0.0%)	
Other hispanic	30 (2.1%)	2 (1.7%)	0 (0.0%)	
Non-hispanic white	863 (60.6%)	84 (73.0%)	14 (87.5%)	
Non-hispanic black	292 (20.5%)	12 (10.4%)	2 (12.5%)	
Other race	45 (3.2%)	1 (0.9%)	0 (0.0%)	
Education level				0.030
Less than 9th grade	146 (10.3%)	20 (17.4%)	1 (6.2%)	
9–11th grade includes 12th grade with no diploma	202 (14.2%)	19 (16.5%)	0 (0.0%)	
High school grad or GED or equivalent	370 (26.0%)	30 (26.1%)	4 (25.0%)	
Some college or AA degree	370 (26.0%)	27 (23.5%)	9 (56.2%)	
College graduate or above	336 (23.6%)	19 (16.5%)	2 (12.5%)	
General health condition				0.756
Excellent	129 (9.2%)	10 (8.7%)	1 (6.7%)	
Very good	430 (30.6%)	39 (33.9%)	5 (33.3%)	
Good	539 (38.4%)	37 (32.2%)	8 (53.3%)	
Fair	252 (18.0%)	23 (20.0%)	1 (6.7%)	
Poor	53 (3.8%)	6 (5.2%)	0 (0.0%)	
Had at least 12 alcoholic drinks per year				0.784
Yes	958 (68.3%)	80 (69.6%)	8 (53.3%)	
No	443 (31.6%)	35 (30.4%)	7 (46.7%)	
Unknown	1 (0.1%)	0 (0.0%)	0 (0.0%)	
Used tobacco/nicotine last 5 days				0.606
Yes	335 (23.9%)	23 (20.0%)	3 (20.0%)	
No	1,067 (76.1%)	92 (80.0%)	12 (80.0%)	
Diabetes				0.420
Yes	181 (12.7%)	17 (14.8%)	0 (0.0%)	
No	1,209 (84.9%)	94 (81.7%)	16 (100.0%)	
Borderline	34 (2.4%)	4 (3.5%)	0 (0.0%)	
HBP				0.045
Yes	646 (45.4%)	66 (57.4%)	8 (50.0%)	
No	776 (54.6%)	49 (42.6%)	8 (50.0%)	
High cholesterol level				0.052
Yes	595 (41.8%)	45 (39.1%)	9 (56.2%)	
No	592 (41.5%)	59 (51.3%)	7 (43.8%)	
Unknown	238 (16.7%)	11 (9.6%)	0 (0.0%)	
DR				0.968
No retinopathy	1,246 (87.4%)	97 (85.1%)	13 (86.7%)	
Mild NPR	149 (10.5%)	14 (12.3%)	2 (13.3%)	
Moderate or severe NPR	27 (1.9%)	3 (2.6%)	0 (0.0%)	
PR	3 (0.2%)	0 (0.0%)	0 (0.0%)	
Cataract operation				<0.001
Yes	152 (10.7%)	30 (26.1%)	10 (62.5%)	
No	1,273 (89.3%)	85 (73.9%)	6 (37.5%)	
Glaucoma				0.021
Yes	79 (5.6%)	13 (11.5%)	2 (12.5%)	
No	1,343 (94.4%)	100 (88.5%)	14 (87.5%)	

## Results

### Participants Selection

**FIGURE 1:**
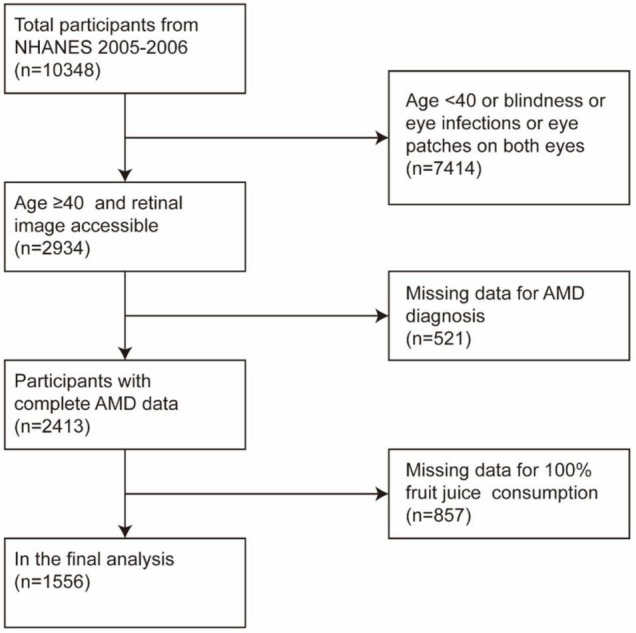
Socio-demographic and clinical characteristics of participants by age-related macular degeneration (AMD) status.

A total of 10,348 participants in the NHANES 2005–2006, 7,414 were excluded due to Age ≤40 years or blindness or eye infections or eye patches on both eyes, and 2,934 were left, of which 521 had missing AMD data and 857 have missing 100% Fruit juice consumption data, and finally 1,556 people conducted data analysis (Figure 1).

### Demographic and Clinical Characteristics of Age-Related Macular Degeneration and 100% Fruit Juice Consumption

Table 1 provides information about the subjects’ demographic characteristics, general health status, BMI and health-related behaviors, systemic comorbidities, eye disease comorbidities, and 100% fruit juice consumption. AMD and late AMD column distribution. The prevalence of any AMD is 8.4%, the prevalence of early AMD is 7.4%, and the overall prevalence of advanced AMD is 1.0%. The average age of patients without AMD was 59.1 years (SE = 12.6), and the average age of patients with early AMD was 70.8 years (SE = 11.4). The average age of patients with advanced AMD was 78.1 years (SE = 7.4). The majority of patients with advanced AMD were women (*n* = 9; 56.2%). The race is mainly non-hispanic white (*n* = 14; 87.5%). Table 2 provides the demographic characteristics, general health status, BMI and health-related behaviors, systemic comorbidities, eye disease comorbidities, and AMD status of the 100% fruit juice consumption no, medium, and high groups.

**TABLE 2:** Socio-demographic and clinical characteristics by 100% fruit juice consumption.

	100% fruit juice consumption			
	
Characteristics	Never	Moderate	High	*P*-value
N	555	926	75	
Age	61.3 ± 13.1	59.4 ± 12.9	60.7 ± 12.6	0.025
PIR	2.9 ± 1.6	2.9 ± 1.6	2.2 ± 1.4	0.003
Body mass index(kg/m)	29.4 ± 7.8	29.1 ± 6.3	29.4 ± 6.7	0.725
ARM				0.048
No ARM	506 (91.2%)	854 (92.2%)	65 (86.7%)	
Early ARM	46 (8.3%)	62 (6.7%)	7 (9.3%)	
Late ARM	3 (0.5%)	10 (1.1%)	3 (4.0%)	
Gender				0.311
Male	284 (51.2%)	480 (51.8%)	32 (42.7%)	
Female	271 (48.8%)	446 (48.2%)	43 (57.3%)	
RACE				0.010
Mexican American	57 (10.3%)	143 (15.4%)	11 (14.7%)	
Other hispanic	9 (1.6%)	20 (2.2%)	3 (4.0%)	
Non-hispanic white	376 (67.7%)	546 (59.0%)	39 (52.0%)	
Non-hispanic black	100 (18.0%)	185 (20.0%)	21 (28.0%)	
Other race	13 (2.3%)	32 (3.5%)	1 (1.3%)	
Education level				0.124
Less Than 9th Grade	54 (9.7%)	102 (11.0%)	11 (14.9%)	
9–11th grade includes 12th grade with no diploma	88 (15.9%)	120 (13.0%)	13 (17.6%)	
High school grad or GED or equivalent	160 (28.8%)	223 (24.1%)	21 (28.4%)	
Some college or AA degree	138 (24.9%)	251 (27.1%)	17 (23.0%)	
College graduate or above	115 (20.7%)	230 (24.8%)	12 (16.2%)	
General health condition				0.241
Excellent	52 (9.5%)	82 (9.0%)	6 (8.0%)	
Very good	177 (32.5%)	280 (30.7%)	17 (22.7%)	
Good	201 (36.9%)	351 (38.4%)	32 (42.7%)	
Fair	103 (18.9%)	158 (17.3%)	15 (20.0%)	
Poor	12 (2.2%)	42 (4.6%)	5 (6.7%)	
Had at least 12 alcoholics drinks per year				0.255
Yes	379 (69.7%)	624 (68.3%)	43 (57.3%)	
No	165 (30.3%)	288 (31.5%)	32 (42.7%)	
Don t know	0 (0.0%)	1 (0.1%)	0 (0.0%)	
Used tobacco/nicotine last 5 days				0.963
Yes	130 (23.9%)	214 (23.4%)	17 (22.7%)	
No	414 (76.1%)	699 (76.6%)	58 (77.3%)	
Diabetes				0.003
Yes	89 (16.0%)	96 (10.4%)	13 (17.3%)	
No	450 (81.1%)	811 (87.7%)	58 (77.3%)	
Borderline	16 (2.9%)	18 (1.9%)	4 (5.3%)	
HBP				0.593
Yes	266 (48.1%)	420 (45.4%)	34 (45.3%)	
No	287 (51.9%)	505 (54.6%)	41 (54.7%)	
High cholesterol level				0.788
Yes	235 (42.3%)	387 (41.8%)	27 (36.0%)	
No	230 (41.4%)	391 (42.2%)	37 (49.3%)	
Unknown	90 (16.2%)	148 (16.0%)	11 (14.7%)	
DR				0.721
No retinopathy	483 (87.0%)	810 (87.6%)	63 (85.1%)	
Mild NPR	56 (10.1%)	99 (10.7%)	10 (13.5%)	
Moderate or severe NPR	14 (2.5%)	15 (1.6%)	1 (1.4%)	
PR	2 (0.4%)	1 (0.1%)	0 (0.0%)	
Cataract Operation				0.002
Yes	90 (16.2%)	93 (10.0%)	9 (12.0%)	
No	465 (83.8%)	833 (90.0%)	66 (88.0%)	
Glaucoma				0.018
Yes	46 (8.3%)	43 (4.7%)	5 (6.7%)	
No	509 (91.7%)	878 (95.3%)	70 (93.3%)	

### The Relationship Between the Consumption of 100% Fruit Juice and the Prevalence of Age-Related Macular Degeneration

**FIGURE 2:**
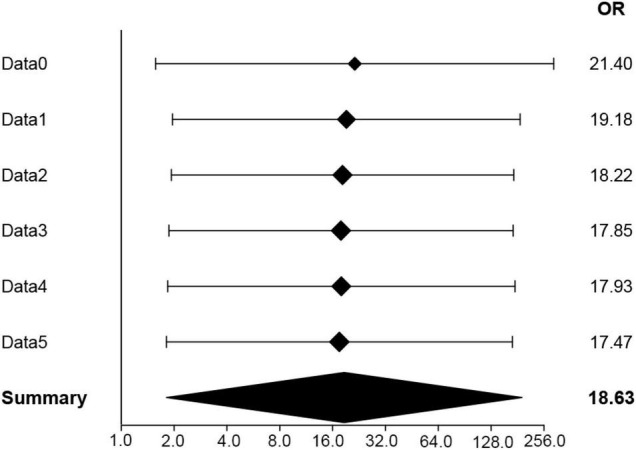
Forest plot of OR value obtained by analysis of original data (Data0) and multiple imputation data (Data1–5).

We constructed logistic regression models and found that 100% fruit juice consumption levels were not statistically correlated with early AMD and any AMD (Tables 3, 4). However, we found that without adjusting for confounding factors, participants with high consumption of 100% fruit juice were likely to be diagnosed with AMD than those who never consumed 100% fruit juice: (OR, 7.78; 95%CI, 1.54–39.37; *p* = 0.0131) (Table 3). After adjusting gender, age, race, education, income and poverty ratio, health-related behaviors: healthy body, BMI, smoking, drinking, systemic diseases comorbidities: diabetes, high blood pressure, hyperlipidemia; ocular complications: glaucoma, cataract surgery history, diabetic retinopathy, the relationship between high consumption of 100% fruit juice and the prevalence of advanced AMD is statistically significant: (OR, 21.40; 95%CI, 1.57–291.10; *p* = 0.0214) (Table 5). The result after combining multiple imputation coefficients: (OR, 18.63; 95%CI, 1.81–191.80; *p* = 0.01395) (Figure 2 and Table 6).

**TABLE 3:** Multi-class logistic regression model of the association between AMD and 100% fruit juice consumption.

	No AMD	Early AMD	Late AMD
		OR (95% CI)	OR (95% CI)
		*P*-value	*P*-value
Never	1.0 (ref.)	0.0909 (0.0672, 0.1229)	0.0059 (0.0019, 0.0184)
		<0.0001	<0.0001
Moderate	1.0 (ref.)	0.7986 (0.5370, 1.1877)	1.9750 (0.5410, 7.2102)
		0.2668	0.3029
High	1.0 (ref.)	1.1846 (0.5134, 2.7332)	7.7847 (1.5390, 39.3774)
		0.6912	0.0131

**TABLE 4:** Multinomial logistic regression model of the relationship between any AMD and 100% fruit juice consumption.

Exposure	Non-adjusted	Adjusted I	Adjusted II
	OR (95% CI)	OR (95% CI)	OR (95% CI)
	*P*-value	*P*-value	*P*-value
**100% fruit juice consumption**			
Never	1.0 (ref.)	1.0 (ref.)	1.0 (ref.)
Moderate	0.9 (0.6, 1.3) 0.474	1.0 (0.7, 1.5) 0.963	1.0 (0.6, 1.5) 0.905
High	1.6 (0.8, 3.3) 0.212	1.9 (0.9, 4.1) 0.110	2.0 (0.9, 4.5) 0.114

*Outcome variate: ARM.*

*Exposure variate: 100% fruit juice consumption.*

*Non-adjusted model adjusts for: none.*

*Adjust I model adjust for: Gender; Age; RACE.*

*Adjust II model adjust for: Gender; Age; RACE; Education Level; PIR; General Health Condition; Body Mass Index (kg/m); Had at least 12 alcoholic drinks per year; Used tobacco/nicotine last 5 days; Diabetes; HBP; High Cholesterol Level; DR; Cataract Operation; Glaucoma.*

**TABLE 5:** The relationship between 100% fruit juice consumption and late AMD.

Exposure	Non-adjusted	Adjusted I	Adjusted II
	OR (95% CI)	OR (95% CI)	OR (95% CI)
	*P*-value	*P*-value	*P*-value
**Pure fruit juice consumption**			
No	1.0	1.0	1.0
Moderate	1.98 (0.54, 7.21)	2.51 (0.67, 9.37)	3.97 (0.76, 20.85)
	0.3028	0.1723	0.1028
High	7.78 (1.54, 39.37)	12.50 (2.18, 71.66)	21.40 (1.57, 291.10)
	0.0131	0.0046	0.0214

*(original data).*

*Non-adjusted model adjusts for: none.*

*Adjust I model adjusts for: Gender; Age; RACE.*

*Adjust II model adjusts for: Gender; Age; RACE; Education Level; PIR; General Health Condition; Body Mass Index (kg/m); Diabetes; HBP; High Cholesterol Level; Had at least 12 alcoholic drinks per year; Used tobacco/nicotine last 5 days; DR; Glaucoma; Cataract Operation.*

*Non-adjusted model adjusts for: None.*

*Adjust I model adjust for: Gender; Age; RACE.*

*Adjust II model adjust for: Gender; Age; RACE; Education Level; PIR; General Health Condition; Body Mass Index (kg/m); Diabetes; HBP; High Cholesterol Level; Had at least 12 alcohol drinks per year; Used tobacco/nicotine last 5 days; DR; Glaucoma; Cataract Operation.*

**TABLE 6:** The relationship between 100% fruit juice consumption and late AMD (data after multi-imputation and emerging).

Exposure	Non-adjusted	Adjusted I	Adjusted II
	OR (95% CI)	OR (95% CI)	OR (95% CI)
	*P*-value	*P*-value	*P*-value
**Pure fruit juice consumption**			
No	1.0	1.0	1.0
Moderate	1.98 (0.54, 7.21)	2.51 (0.67, 9.37)	3.97 (0.76, 20.85)
	0.3028	0.1723	0.1028
High	7.78 (1.54, 39.37)	12.50 (2.18, 71.66)	18.63 (1.81,191.80)
	0.0131	0.0046	0.01395

## Discussion

Age-related macular degeneration is one of the global blinding eye diseases. Our study tried to find ways for preventing AMD from the perspective of dietary habits. In our study, the prevalence of advanced AMD was approximately 1.0% (*n* = 16) after screening the subjects. The results are roughly consistent with the original NHANES data, and the prevalence of advanced AMD was 1.1% (*n* = 27). Meanwhile, it is similar to the prevalence of advanced AMD reported by Klein: 0.8% (95%CI 0.5–1.3) (4). Our study found that participants with high consumption of 100% fruit juice were likely to be diagnosed with advanced AMD after adjusting for various confounding factors. Provide clues to decrease the prevalence of advanced AMD from the perspective of dietary habits. We did not find a relationship between 100% fruit juice consumption and early AMD or any AMD, nor did we find a statistical association between moderate 100% fruit juice consumption and AMD.

Age-related macular degeneration is a multifactorial disease, and its pathogenesis involves disorders of complement, lipids, angiogenesis, inflammation, and extracellular matrix pathways. The high-risk factor for AMD is the growth of age. With age, the lipids deposited on Bruch’s membrane of the retina increase, and the deposited lipids inhibit the nutrient exchange of RPE, the antioxidant enzymes of the retina decrease, and the retina is susceptible to oxidative stress and free radical damage, leading to the AMD development (17). People with non-proliferative or proliferative diabetic retinopathy have a higher risk of developing AMD than those without diabetes or diabetic retinopathy (18). The increased risk of AMD (especially in advanced AMD) is dose-dependent with overweight, and maintaining normal body weight and avoiding weight gain may be potential protective factors (19). Metelitsina et al. (20) reported that choroidal blood flow is lower in AMD patients with systemic hypertension than in AMD patients without systemic hypertension, and the reduction in choroidal blood circulation may help explain the mechanism by which systemic hypertension promotes AMD progression and choroidal neovascularization.

Whether drinking 100% pure fruit juice affects health has always been controversial. Some researchers believe that drinking 100% fruit juice has nothing to do with disease. In addition to increasing the risk of dental caries in children and slightly increasing the weight of young children and adults, there is no clear evidence that drinking 100% fruit juice can adversely affect the health (8). Agarwal et al. reported that 100% fruit juice intake is correlated with healthier diet quality and increase intake of nutrients, such as energy, calcium, magnesium, potassium, vitamin C and vitamin D. Substituting 100% fruit juice intake for whole fruits has no significant effect on nutrients other than dietary fiber (21). Previous studies found that 100% fruit juice consumption does not affect adult blood pressure, fasting blood glucose, total cholesterol, HDL high-density lipoprotein cholesterol, and LDL low-density lipoprotein cholesterol levels (22) 100% fruit juice does not affect the changes of alanine aminotransferase (23). Some researchers are cautious about the consumption of 100% fruit juice: 100% fruit juice is rich in natural sugars (24, 25). In the United States, the per capita daily calorie intake has increased due to the intake of sugar-sweetened beverages and 100% fruit juice (26). An energy-intensive diet destroys the body’s ability to accurately estimate energy requirements and energy intake (27), which increases calorie intake (28). Although 100% fruit juices contain most of the nutrients of whole fruits, they contain no or very little fiber (29). 100% fruit juice has moderately high glycemic indexes (30). Randomized controlled trials have shown that there is a difference in the way the body records solid sugar calories and liquid sugar calories. Liquid sugar calories consume more free energy than solid sugar calories (30, 31). Studies have shown that 100% fruit juice has an adverse health effect similar to sugar-sweetened beverages (13, 32). Shefferly et al. showed that high consumption of 100% fruit juice at the age of 2 is correlated with an increase chance of being overweight at the age of 2–4 (13). A meta-analysis of two prospective cohort studies showed that drinking 100% fruit juice is associated with a slight increase in the risk of diabetes in adults (relative risk < 1.1) (9, 33). Auerbach et al. reported that daily intake of ≥24 ounces of 100% fruit juice is correlated with a higher risk of hypertension (34). 100% fruit juice is considered to be a high-sugar and high-calorie food that increases the risk of obesity in preschool children (11). A cross-sectional study reported that consumption of juice >12 ounces per day would increase the possibility of short stature and obesity (10). Wojcicki et al. recommend limiting or removing 100% fruit juice intake from the diet of children (35). Nunes and Anastasiou (36) reported that high fructose corn syrup dietary increases intestinal surface area, leading to increased absorption of dietary nutrients and weight gain. Although the concept of 100% fruit juice is different from sweetened beverages, the two have the same thing: 100% fruit juice contains more sugar per ounce than ordinary soda water (25). A previous study reports that in the United States, young people with type 1 diabetes consuming at least one cup of diet soft drink a day have 0.4% higher levels of glycosylated hemoglobin than consuming less than one cup of hemoglobin per day, with 4 mg/dL higher TC and 6 mg/dL higher TGs (37). In the North Manhattan study, people who drank diet soft drinks every day had an increased risk of myocardial infarction, stroke, and vascular death compared to those who did not drink diet soft drinks. However, ingesting a small number of soft drinks did not enhance the risk of vascular events (38). Similar to our study, the intake of a moderate amount of 100% fruit juice is not correlated with the risk of AMD, but daily intake of 100% fruit juice increases the risk of developing advanced AMD than those who never drink it.

This study has the following limitations: (a) NHANES used a cross-sectional study design. We can only explore the correlation and cannot further explore the causality. The cross-sectional design means that we cannot accurately grasp the 100% fruit juice consumption data when diagnosing AMD, because most people may change their eating habits due to various reasons. To tackle the limitations of cross-sectional research design, further longitudinal design research is needed. (b) Confidence interval refers to the estimated interval of the overall parameter constructed by the sample statistics. Compared with the wide confidence interval, the narrow confidence interval can provide more information about the overall parameter. However, small sample size may result in a wide confidence interval. For example, Qiu et al. (39) reported that compared with emmetropia, the probability of visual field defects in high myopia is significantly increased (OR, 14.43; 95% CI, 5.13–40.61). In our study, only the 2005–2006 NHANES can provide 100% fruit juice consumption data and AMD staging data simultaneously, and the prevalence of advanced AMD is only about 1.1%, resulting in only 16 cases of advanced AMD, which is relatively small. The small sample size resulted in a large confidence interval for the OR value. However, regardless of whether the imputation data or the original data is used in the regression analysis, whether the covariates are adjusted, the direction of the OR results is not affected, and it does not affect the conclusion that a high intake of 100% pure juice increases the risk of advanced AMD. As for how much high consumption of 100% fruit juice influence the risk of advanced AMD compared with those who never drink, more samples are needed to support the data. (c) The acquisition methods of outcome variables, exposure variables and covariates can be more precise and accurate. Although we use a well-validated FFQ to collect our soft drink data. The consumption of 100% fruit juice was assessed based on an interview in which participants were asked to recall their food consumption patterns over the past year. Because of the long-time gaps, food consumption patterns are likely to change during these periods. Therefore, the consumption frequency of 100% fruit juice provided in the interview may be biased. We study the cross-sectional nature, meaning that we do not know if the current personal consumption before diet drinks beverage ordinary consumers may change their way of life during diagnosis of Dr. Although we adjust dietary habits of the change in the past 5 years, it will not be captured in the diagnosis of diabetes or Dr. Diet soft drink consumption changes, this is when most patients are likely to implement diet or changes in lifestyle. Hence, our study may overestimate the association between diet soft drink consumption and DR, and our conclusions should be inferred with this limitation in mind. Longitudinally design studies are needed to tackle the limitations of cross-sectional study design. As for the consumption variable of 100% fruit juice, the specific fruit juice types and composition are not clear. We found that the association between self-reported 100% fruit juice intake and AMD may be explained by several other underlying conditions associated with self-reported 100% fruit juice intake and AMD. It is also possible that the link was discovered accidentally. Until this association is evaluated in further prospective studies, we would not advise patients to discontinue 100% fruit juice to reduce their risk of developing advanced AMD, especially given the known benefits of 100% fruit juice for other medical conditions. Although AMD was detected by grading digital images of the fundus using a standard grading system, no additional data such as OCT, angiography, etc., were used in the NHANES to verify the subject’s diagnosis of AMD, only based on retinal images. The number of AMD patients may have been underestimated because some fundus images were excluded from the survey due to poor image quality (*n* = 224) or non-presence of images (*n* = 969). In addition, only 1.1 percent of the study population had advanced AMD. Such a small number of advanced AMD cases may result in poor statistical outcomes to detect an association between 100% fruit juice intake and advanced AMD. However, the prevalence of late and early stage AMD was similar to other population-based studies. (d) NHANES adapted a stratified multi-stage sampling design and requires a weighted scheme to precisely determine the prevalence of disease in the U.S. population. Because we focused on the relationship between 100% fruit juice consumption and the risk of AMD, so we screened the research subjects and did not weigh the data when studying the correlation in some high-grade literatures. Therefore, weighted data was not used in our data analysis. (e) Despite adjustment for some confounders, it was not possible to exclude all risks of bias and possible incidental findings. Unmeasured or residual confounders may lead to additional analysis bias. We cannot rule out the possibility of statistical chance.

## Conclusion and Prospect

To sum up, we found that moderate consumption of 100% fruit juice did not affect AMD. After adjusting for various confounding factors, participants with high consumption of 100% fruit juice were more likely to be diagnosed with advanced AMD than participants who self-reported that they never consumed 100% fruit juice. Therefore, the increased prevalence of advanced AMD may be due to the adverse effects of high-frequency consumption of 100% fruit juice, rather than the unhealthy nature of 100% fruit juice itself. Khan et al. called for consideration of the dose-response relationship when studying the relationship between 100% fruit juice consumption and disease (40). There is no significant statistical relationship between the moderate consumption of 100% fruit juice and AMD, while high consumption significantly increases the risk of advanced AMD. It can discourage excessive drinking of 100% juice to avoid increasing the possibility of advanced AMD. Because our study has limitations, including limited sample size, unclear fruit types and specific components of 100% fruit juice, larger sample size and more detailed researches are needed to further explore the association between consumption of 100% fruit juice and AMD. The study on the relationship between age-related macular degeneration and the specific dosage ranges of 100% fruit juice will provide more evidence-based evidence for dietary guidelines and public policies.

## Data Availability Statement

The original contributions presented in the study are included in the article/supplementary material, further inquiries can be directed to the corresponding author.

## Author Contributions

MS wrote the draft of the manuscript. BC contributed to the manuscript revision. Both authors contributed to data analysis and approved the submitted version.

## Conflict of Interest

The authors declare that the research was conducted in the absence of any commercial or financial relationships that could be construed as a potential conflict of interest.

## Publisher’s Note

All claims expressed in this article are solely those of the authors and do not necessarily represent those of their affiliated organizations, or those of the publisher, the editors and the reviewers. Any product that may be evaluated in this article, or claim that may be made by its manufacturer, is not guaranteed or endorsed by the publisher.
